# Minimally invasive removal of apocrine glands: a study of 100 cases using three maneuvers with blunt scissors

**DOI:** 10.1093/jscr/rjad337

**Published:** 2023-06-12

**Authors:** Wen-Tsao Ho

**Affiliations:** Department of Dermatology, Ho Wen Tsao Skin Clinic, New Taipei City 244, Taiwan

## Abstract

A case series study describes a minimally invasive surgical technique for removing axillary apocrine glands using blunt scissors. The technique involved making two small incisions, removing the glands using three maneuvers, and evaluating patient satisfaction and post-surgery complications. Of 100 patients, 92% reported being satisfied with the results, with no reported complications. The study suggests this technique is safe and effective, offering a minimally invasive alternative to traditional surgery with fewer negative cosmetic outcomes. However, further research is needed to assess its long-term efficacy and safety.

## INTRODUCTION

Osmidrosis surgery, which involves the removal of axillary apocrine glands, is a common surgical procedure for severe cases [1, 2]. Traditionally, long incisions have been utilised for osmidrosis surgery, which can be made very cleanly with scissors. Although traditional surgery provides better visibility and can result in thorough clearance, it often results in relatively long scars that may negatively impact cosmetic outcomes. Surgeons are investigating various surgical methods for removing axillary apocrine glands, including traditional techniques, liposuction-assisted curettage and scraping [3–5]. Conversely, making very small incisions may sacrifice the clarity of view, and minimal incisions may not have the stretchability required to clear large areas [6–8]. Scissors are often limited by space and are not very useful in these situations, leading to suboptimal surgical outcomes. In this study, we aimed to combine three new maneuvers that enable the effective use of scissors in small incisions for osmidrosis surgery, in order to improve surgical outcomes and minimize negative cosmetic outcomes.

## CASE SERIES

### Material and methods

We recruited 100 patients (80% female and 20% male) aged between 20 and 45 years who had axillary malodor.

### Procedure

Patients were positioned in a supine position with the affected arm abducted at approximately 90 degrees. Prior to surgery, axillary hair was shaved clearly. Tumescent anesthesia was administered for diffuse infiltration and to aid in the undermining process. Each axillary region was infiltrated subcutaneously with a solution of 200-mL normal saline, 1.0-mL 1:1000 epinephrine and 20-mL 2% lidocaine for anesthesia and vasoconstriction to minimize bleeding during the procedure.

### Surgical technique

**Figure 1:**
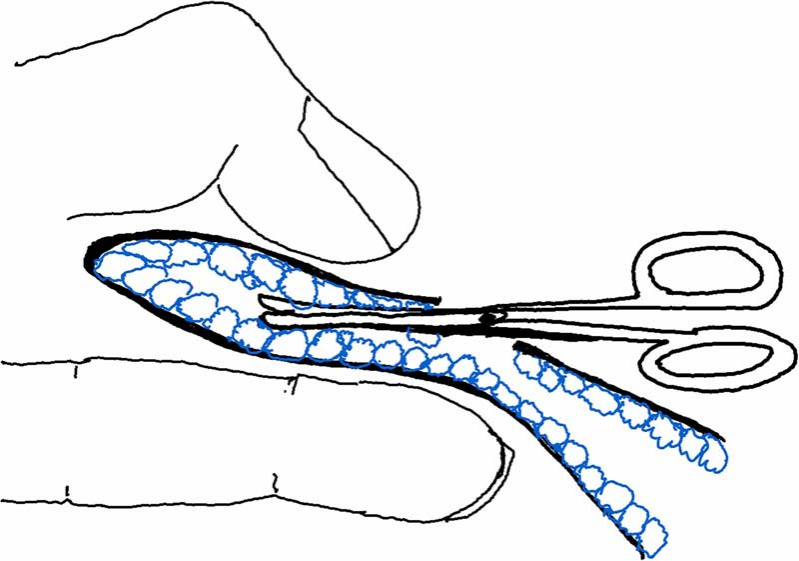
Illustration of Maneuver 1. Small, blunt scissors inserted into the skin flap opening lifted by the assisting hand. Scissors used to cut apocrine glands between flaps.

Two small incisions, each measuring 5 mm, were made at the axillary boundary of each of the three equal parts of the axilla. The apocrine glands were surgically removed using blunt scissors. Three different techniques were used to remove the glands:

Maneuver 1 involved gently pulling the skin flap upwards using the index and thumb of the assisting hand, and inserting the small blunt scissors into the opening. The narrowest part of the scissors’ cross was kept at the opening, whereas the scissors were positioned in the middle of both sides of the skin flap. The apocrine glands were blindly cut by repeatedly opening and closing the scissors while changing positions (see Fig. 1) (Video 1).

**Figure 2:**
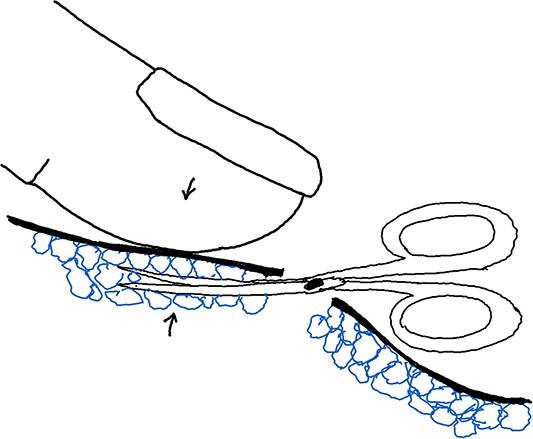
Illustration of Maneuver 2. Skin flap pressed down with assisting hand’s fingers. Blunt scissors inserted, supporting flap and cutting glands gently by opening and closing.

Maneuver 2 involved pressing down the skin flap using the assisting hand’s index and middle fingers, and inserting the small blunt scissors into the opening. The narrowest part of the scissors’ cross was positioned from the opening to the small incision. The scissors supported the skin flap upwards (opposite to the assisting hand), and the glands were cut by gently opening and closing the scissors repeatedly (see Fig. 2) (Video 2).

**Figure 3:**
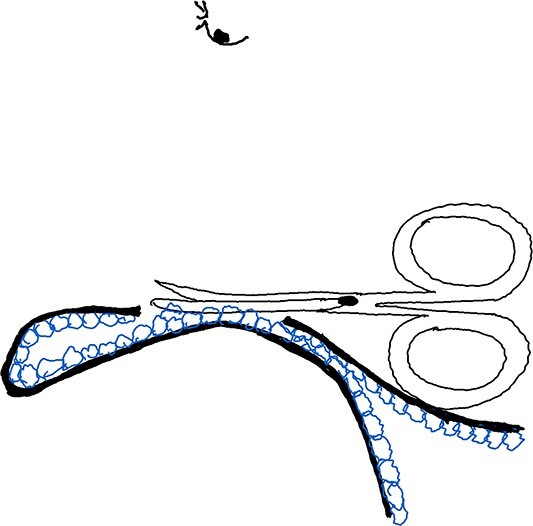
Illustration of Maneuver 3. Skin flap pulled up by the assisting hand, creating a small space to expose and remove apocrine glands under visual guidance using a pinch and turn over technique.

Maneuver 3 involved pulling the skin flap upwards using the thumb and index finger of the assisting hand, whereas the same hand’s thumb supported the opening to create a small space, exposing the apocrine gland for direct cutting. The glands were removed by constantly changing positions and cutting under visual guidance (pinch and turn over) (see Fig. 3) (Video 3).

At last, we will check up the clearance through the small incisions as demonstrated in Video 4.

After the apocrine glands were completely removed, and the axillary wound was covered with a bulky, ball-shaped gauze, about the size of a fist, which was fixed in place with sutures to firmly press the skin flap and subcutaneous skin together (tie-over dressing). The procedure was performed on an outpatient basis, and patients returned after 3 days for tie-over dressing and suture removal.

### Outcome measures

The extent of flap erosion or necrosis was evaluated post-surgery by classifying it into one of three categories: mild, moderate or severe after 1 week. The efficacy of the surgical intervention was evaluated by assessing patient satisfaction with malodor elimination after 6 months. Patients rated their level of satisfaction on a five-point scale ranging from very satisfied to very unsatisfied.

## RESULTS

A total of 100 patients were enrolled in the study, with 80% being female and 20% being male. The age of the patients ranged from 20 to 45 years, with a mean age of 32 years (see Table 1).

**Table 1:** Demographic characteristics of the study population

**Characteristic**	**Number**	**Percentage (%)**
Total	100	100
Female	80	80
Male	20	20

### Wound healing

Out of the 100 patients, 72 had normal to mild epidermal damage, 24 had moderate epidermal damage and 4 had severe epidermal damage. The severity of damage was not significantly correlated with the age or sex of the patients (see Table 2).

**Table 2:** Wound healing and patient satisfaction

**Outcome**	**Number**	**Percentage (%)**
Wound healing		
Normal to mild damage	72	72
Moderate damage	24	24
Severe damage	4	4
Patient satisfaction		
Very satisfied	66	66
Satisfied	26	26
Average satisfaction	8	8
Unsatisfied/very unsatisfied	0	0

### Surgical efficacy

Overall, 92% of the patients reported being satisfied with the results of the surgery. Specifically, 66 patients reported being very satisfied, 26 were satisfied and 8 reported average satisfaction. None of the patients reported being unsatisfied or very unsatisfied. There was no significant difference in the satisfaction rate between male and female patients (see Table 2).

### Complications

No hematoma was noted in any of the patients.

## DISCUSSION

The surgical technique of removing apocrine glands using blunt scissors is a simple and effective method for treating axillary malodor. The results of this study suggest that this technique can be an alternative to other surgical methods.

Traditional surgical procedures for treating axillary osmidrosis involve making a relatively large incision and carefully dissecting and removing the affected tissue within the field of view. However, making the incision smaller to reduce scarring may compromise the thoroughness of the removal process and make it more difficult to achieve complete removal of the affected tissue. Many studies have explored various techniques to reduce the incision size and increase clearance rates for apocrine gland removal. These techniques include the use of devices such as liposuction-assisted curettage, blind trimming with ophthalmic scissors, fenestrated cup curette, CO2 laser and subcutaneous tissue removal [8–12]. In our study, we aimed to evaluate the effectiveness of three specific maneuvers that could achieve results comparable with those obtained with liposuction-assisted curettage.

Hu *et al*. describe a surgical technique that involves making mini-incisions in the axilla and using liposuction-curettage to remove the apocrine glands [12]. A study involving 449 axillae found that patients who underwent well-organised liposuction-curettage and received appropriate post-operative care experienced excellent elimination of malodor in 89.81% of cases. However, skin necrosis occurred in 2.55% of cases, and 17.83% experienced local damage to the epidermis. Wu describes a study involving 156 patients who underwent suction-assisted cartilage shaver under local anesthesia on an outpatient basis [13]. Patients were followed for an average of 16 months, and the total satisfaction rate was 97.4%. The patient complication rate was 7.7%, and the wound complication rate was 5.1%. Tseng *et al.* evaluated the effectiveness of a modified cartilage shaving procedure for treating axillary osmidrosis. The study found that 92.3% of patients achieved excellent-to-good results, 5.1% had acceptable results and 2.6% had fair results [14]. Huang *et al*. reported before treatment, the mean DLQI score was 11.3, but after the procedure, the mean score decreased significantly to 0.8, indicating a 93% reduction. Additionally, 84.2% of patients reported a 90% or greater reduction in odor, and 34.3% were greatly satisfied, with 58.6% reporting absolute satisfaction with the procedure [15].

Based on the available data, the satisfaction rates reported in both the suction-assisted cartilage shaver study and my scissor maneuvers study were comparable. Suction-assisted cartilage shaver has shown promising results as a treatment for removing apocrine glands, with benefits including a short operation time around 50 min, inconspicuous scarring and a speedy recovery that allows patients to quickly resume their daily activities [16]. However, it should be noted that the operation time in my study was longer, with an average of 2 h compared to the reported 50-min operation time in the suction-assisted cartilage shaver study. However, it is important to consider that the operation time may depend on the surgeon’s level of experience. With more experience, the operation time may become shorter. I have attempted to integrate two surgical techniques by utilising a suction-assisted cartilage shaver to remove half of the thickness of the glands, followed by the use of small scissors or a combination of the two methods to remove the remaining ones. This approach seems to increase clearance rates and reduce the surgical time. As the shaver always blindly removes tissue, there is always the possibility of dead corners, and the skin flap may be shaved too thin, leading to complications. Using small scissors can loosen the fibrotic bands and ensure a cleaner removal, whereas the two techniques complement each other.

My scissor maneuvers 1 and 2 involve blindly cutting the apocrine glands, requiring careful maneuvering to avoid harming the skin flap. These maneuvers can be performed safely with proficiency, but inexperienced operators must be cautious not to damage the skin flap. In contrast, maneuver 3 not only allows for more thorough removal within the visual field but also facilitates the final confirmation of complete removal.

Creating two incisions during a procedure can provide a number of benefits. When working with a skin flap that has limited stretchability, making two incisions can help expand the field of view, making it easier to see what’s going on. This is especially useful at the end of the procedure when it’s important to check for any remaining apocrine glands. By flipping over the skin flap randomly and inspecting it carefully (a technique known as pinch and turn over), surgeons can ensure that they haven’t missed anything important. When cutting out apocrine glands under direct vision, it is important to be careful not to damage the dermis, which contains a network of small blood vessels. Cutting too close to the dermis could theoretically result in necrosis of the skin flap.

In contrast, using a single small incision and blindly scraping around is not as effective, as it can be difficult to see everything that needs to be removed. Our modification of creating two incisions is particularly helpful, as it allows for a more thorough removal of apocrine glands. The final pinch and turn-over technique is an important part of this process, as it ensures that everything has been removed as thoroughly as possible.

In conclusion, the surgical technique of removing apocrine glands using blunt scissors is a simple and effective method for treating axillary malodor.

## Supplementary Material

maneuver_1_rjad337

maneuver_2_rjad337

maneuver_3_rjad337

check_up_clearance_rjad337
